# Food availability, accessibility and dietary practices during the COVID-19 pandemic: a multi-country survey

**DOI:** 10.1017/S1368980021000987

**Published:** 2021-05

**Authors:** Ali Jafri, Nonsikelelo Mathe, Elom K Aglago, Silvenus O Konyole, Moussa Ouedraogo, Keiron Audain, Urbain Zongo, Amos K Laar, Jeffrey Johnson, Dia Sanou

**Affiliations:** 1 Mohammed VI University of Health Sciences, Université Mohammed VI des Sciences de la Santé, avenue Taieb Naciri, Casablanca, Morocco; 2 Alliance for Canadian Health Outcomes Research in Diabetes, University of Alberta, Alberta, Canada; 3 Nutrition and Metabolism Section, International Agency for Research on Cancer (IARC), Lyon, France; 4 Department of Nutritional Sciences, Masinde Muliro University of Science and Technology, Kakamega, Kenya; 5 Joseph Ki-Zerbo University, Ouagadougou, Burkina Faso; 6 Department of Food Science and Nutrition, University of Zambia, Lusaka, Zambia; 7 University of Ghana, Accra, Ghana; 8 Food and Agriculture Organization of the United Nations, Addis Ababa, Ethiopia

**Keywords:** COVID-19, Food availability, Accessibility, Nutrition security, Coping mechanisms

## Abstract

**Objective:**

To investigate the perceived effects of the coronavirus disease (COVID-19) pandemic lockdown measures on food availability, accessibility, dietary practices and strategies used by participants to cope with these measures.

**Design:**

We conducted a cross-sectional multi-country online survey between May and July 2020. We used a study-specific questionnaire mainly based on the adaptation of questions to assess food security and coping strategies from the World Food Programme’s ‘Emergency Food Security Assessment’ and ‘The Coping Strategy Index’.

**Setting:**

The questionnaire was hosted online using Google Forms and shared using social media platforms.

**Participants:**

A total of 1075 adult participants from eighty-two countries completed the questionnaire.

**Results:**

As a prelude to COVID-19 lockdowns, 62·7 % of the participants reported to have stockpiled food, mainly cereals (59·5 % of the respondents) and legumes (48·8 %). An increase in the prices of staples, such as cereals and legumes, was widely reported. Price increases have been identified as an obstacle to food acquisition by 32·7 % of participants. Participants reported having lesser variety (50·4 %), quality (30·2 %) and quantity (39·2 %) of foods, with disparities across regions. Vulnerable groups were reported to be facing some struggle to acquire adequate food, especially people with chronic diseases (20·2 %), the elderly (17·3 %) and children (14·5 %). To cope with the situation, participants mostly relied on less preferred foods (49 %), reduced portion sizes (30 %) and/or reduced the number of meals (25·7 %).

**Conclusions:**

The COVID-19 pandemic negatively impacted food accessibility and availability, altered dietary practices and worsened the food insecurity situation, particularly in the most fragile regions.

In March 2020, the WHO declared the novel coronavirus outbreak a pandemic. This pandemic started in December 2019 as a local public health issue in the city of Wuhan in China and quickly escalated to become an unprecedented global health situation. Responding to the pandemic, most countries instituted strict measures such as lockdowns which included self-isolation, quarantine, confinement and/or curfews to mitigate the spread of the disease.

As shown by previous experiences, global and regional emergencies can impact food and nutritional security in households and communities. In 2008, following the global economic crisis, food prices increased and created economic and social instabilities in several regions in the world, including higher income countries^(1)^. In 2014, the Western African Ebola virus disease outbreak had a significant impact on food security in most of the affected countries^(2)^. Countries with strong and sustainable food systems usually recover from these crises. However, for those already faced with hunger and socio-economic challenges, the struggle can be more permanent^(3)^.

The relationship between diet-related diseases and food insecurity has been thoroughly investigated^(4,5)^. Individuals from food-insufficient households have significantly higher odds of having poor functional health, restricted activity and multiple chronic conditions (e.g. obesity, heart disease, diabetes and high blood pressure)^(6–8)^. Also, food insecurity has been associated with major depression, distress and having poor social support^(9,10)^. Furthermore, food insecurity has been consistently associated with health problems in young children and toddlers^(11,12)^.

Since the beginning of the COVID-19 pandemic, lockdown measures have disrupted food systems globally and altered availability and access to healthy foods^(13)^. Early reports warned about the potential repercussions of interrupted food chains^(14,15)^. Lockdowns have been slowing harvest and impacting food industries, including the closure of businesses, burying perishable food products or dumping milk by farmers^(16)^. As a result of the supply chain disruption and the increasing consumer demand, the prices of basic foods have begun to rise^(16)^, which could negatively affect people’s access to healthy foods and contribute to food insecurity and malnutrition^(16)^. Furthermore, the unavailability or reduced affordability of healthy foods might lead to greater consumption of ultra-processed and canned foods, which, combined with reduced physical activity, could worsen obesity and other diet-related non-communicable disease.

While there is no doubt that COVID-19 lockdown measures might affect food security and nutrition, the implications of these lockdowns on the availability and accessibility of healthy food worldwide are poorly documented. Therefore, it is imperative to document the impacts of COVID-19 lockdowns on food security, including availability, accessibility and coping mechanisms, in different regions of the world. The current study was undertaken to investigate the perceived effects of COVID-19 lockdown measures on food availability and accessibility and key strategies that participants used to cope with those measures.

## Methodology

### Development of the questionnaire

The data used in the current study were collected through an online questionnaire. The questionnaire was developed using existing assessment tools and drawing on the expertise of the research team. The questionnaire (in English) was adapted from the World Food Programme’s Emergency Food Security Assessment^(17)^ and the Coping Strategy Index^(18)^ and incorporated questions about food access, dietary changes and coping mechanisms during the COVID-19 pandemic. Then, the draft questionnaire was sent electronically to ten experts in nutrition, food science, public health and epidemiology to check the relevance, format and order of the questions. The team of experts were based in Burkina Faso, Canada, Ethiopia, France, Kenya, Morocco and Zambia. The final version of the questionnaire was translated into French and Arabic. It was structured to document the following areas: (i) government confinement and lockdown measures; (ii) food access; (iii) dietary and nutritional changes and (iv) socio-demographic information of the respondents.

### Piloting and sampling

The questionnaire was created and administered via Google Forms. The questionnaire was sent electronically to a small number of volunteers from various countries (*n* 25) so that we may verify the content’s validity of the questions and make amendments to the wording and positioning of the questions. In addition, the study team and the volunteers explored the sensitivity of the questions regarding specific dietary and lifestyle practices such as alcohol consumption, smoking and physical activity.

The link to the finalised survey was shared widely using diverse digital tools including email, Twitter, Facebook, WhatsApp and Reddit. In order to maximise participation and increase the response rate, a number of strategies were used to spread the information regarding the survey, particularly via national and international nutrition societies and networks. A bulk email was circulated to members with an active email account containing a link to the online survey. A reminder notice was sent via email in the following weeks to increase the response rate.

### Data management

Coded data with no personal identifier were collected, exported and stored in password-protected computers. The data were used only for the intended research purposes and only the research team had access to it. The team checked the levels of completion by region and used this data to decide whether focus should be put on specific regions to recruit more participants.

### Data analysis

Descriptive statistics were used to characterise the responses. Responses were analysed by geographical subregions. All the statistical analyses were performed using Stata 14.0 (StataCorp.).

## Results

From May to June 2020, a total of 1075 respondents from eighty-two countries completed the questionnaires in English (62·4 %), in French (36·0 %) and in Arabic (1·65 %). Respondents with missing entries (*n* 13) and duplicates (i.e. repeat respondents) (*n* 33) were excluded from the analysis.

Table 1 summarises the characteristics of the 1029 respondents. The largest number of respondents were from West Africa (24·4 %), followed by West Europe (16·7 %), East Africa (14·2 %), North Africa (13·2 %), North America (10·2 %) and Southern Africa (8·07 %). Respondents had a median age of 35 years, were mostly living in urban areas (83·4 %), were married (51·8 %), had graduate education (84·7 %) and female respondents represented 52·9 %. A total of 85 % of the respondents were employed, while 7·2 % were students. Respondents in North America reported the highest loss of jobs due to the COVID-19 pandemic (5·7 %).

**Table 1:** General characteristics of the study participants (*n* 1029)

	North Africa	West Africa	East Africa	Southern Africa	Western Europe	North America	Other	Total
Total (*n*)	136	251	146	83	172	105	136	1029
Gender (%)								
Female	63·2	37·5	49·3	78·3	53·5	62·0	51·5	52·9
Male	35·3	59·8	50·0	21·7	42·4	34·3	47·1	44·9
Undisclosed	1·47	2·79	0·68	0	4·07	3·81	1·47	2·14
Location (%)								
Rural	2·21	6·37	17·1	4·82	19·8	23·8	13·2	12·2
Suburban	1·47	1·59	2·05	3·61	4·65	18·1	3·68	4·28
Urban	96·3	92·0	80·8	91·6	75·6	58·1	83·1	83·6
Age								
1st quartile	27·0	30·0	28·0	28·0	28·0	33·0	30·0	29·0
Median	33·0	34·0	34·0	37·0	33·0	40·0	35·0	35·0
3rd quartile	40·5	41·0	42·0	42·0	40·5	48·0	40·0	42·0
Education (%)								
Primary	0	0·4	1·37	0	0·59	0	0	0·39
Secondary	3·68	4·78	4·11	9·76	11·2	11·5	5·15	6·73
University degree	31·6	27·5	50·7	53·7	42·9	47·1	39·0	39·5
Master or PhD	64·7	67·3	43·8	36·6	45·3	41·4	55·9	53·4
Employment (%)								
Student	14·0	5·98	3·42	2·41	12·8	3·81	5·15	7·19
Employed	80·2	89·6	84·3	89·2	79·1	78·1	89·7	84·7
Retired	2·21	0·4	2·05	3·61	2·33	4·76	2·21	2·14
Unemployed	1·47	3·59	7·53	4·82	5·23	7·62	2·21	4·47
Unemployed due to COVID	2·21	0·4	2·74	0	0·58	5·71	0·74	1·55
Household income in USD								
First quartile	652·5	200	200	300	2000	3150	420	392·5
Median	1600	500	590	1400	3600	8250	1000	1300
Third quartile	3000	1000	1500	2500	8000	32 500	3000	3900
COVID-19 security measures								
Travel ban (%)	97·8	94·4	85·6	100	83·7	74·3	70·6	87·1
Lockdown (%)	81·6	80·1	86·3	81·9	14·0	12·4	33·8	57·2
Social distancing (%)	94·9	96·4	96·6	95·2	98·8	100	98·5	97·2
Home confinement (%)	95·6	49·4	51·4	89·2	77·3	56·2	69·9	67·1
Closed fast-foods (%)	80·2	57·0	48·6	72·3	73·3	58·1	53·7	62·5
Closed supermarkets (%)	7·35	37·9	6·85	12·1	4·65	5·71	14·7	15·5

Respondents reported that various security measures were in effect in their respective locations, and most (62·7 %) reported that they stockpiled food in anticipation of food shortages during the COVID-19-related lockdowns (Table 2). Cereals were the most stockpiled items (59·5 %), followed by legumes (48·8 %) and canned goods (38·8 %). Perishables like fruits and dairy products were stockpiled by fewer people. Geographically, more people from North America stockpiled food overall (76·2 %), especially canned goods (66·7 %). More people from West and East Africa particularly stockpiled cereals (77·7 and 74·7 %, respectively).

**Table 2:** Relative frequencies of respondents who stockpiled food during the COVID-19 pandemic during April and May 2020

	North Africa	West Africa	East Africa	Southern Africa	Western Europe	North America	Other	Total
Total (*n*)	136	251	146	83	172	105	136	1029
Purchased more food due to COVID-19 (%)	41·9	71·3	67·1	61·5	61·1	76·2	55·2	62·7
Stockpiled cereals (%)	50·7	77·7	74·7	39·8	40·1	56·2	57·4	59·5
Stockpiled meat (%)	29·4	42·2	36·3	45·8	22·7	43·8	36·0	36·1
Stockpiled dairy (%)	18·4	45·4	16·4	14·5	8·72	20·0	30·9	24·6
Stockpiled legumes (%)	43·4	55·0	69·2	37·4	35·5	48·6	44·9	48·8
Stockpiled fruits (%)	15·4	20·3	23·3	22·9	7·6	30·5	19·1	19·1
Stockpiled vegetables (%)	21·3	42·2	29·5	33·7	14·0	37·1	30·9	30·2
Stockpiled canned products (%)	35·3	39·8	16·4	41·0	44·8	66·7	33·8	38·8
Stockpiled sweets (%)	19·1	18·7	13·7	15·7	14·5	24·8	19·9	17·9

**Fig. 1:**
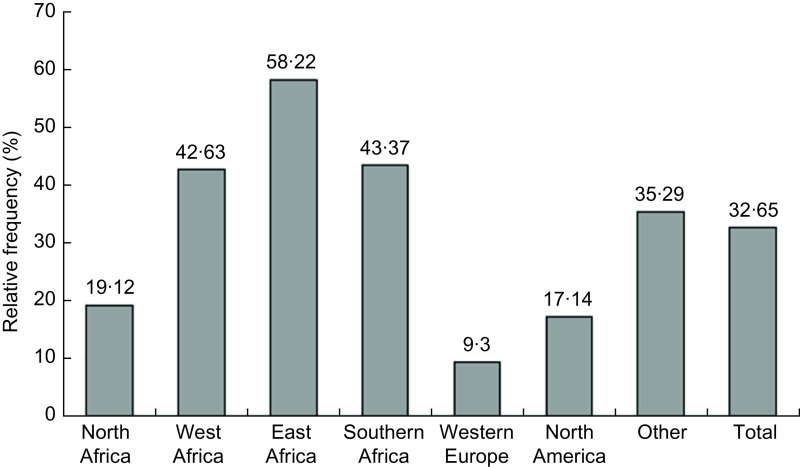
Relative frequency of people whose food acquisition has been affected by price increases during COVID-19

Respondents reported that the prices of food items increased during the lockdowns; the most commonly affected food groups were fruits and vegetables, cereals and meat (Table 3). Increased prices of staples like cereals were most commonly reported in African countries; 77·4 % in East Africa, 61·4 % in West Africa and 54·2 % in Southern Africa. Price increases have been identified as an obstacle to food acquisition by 32·7 % of respondents (Fig. 1). The impact of food price increase on food acquisition was more commonly reported by respondents in African countries, especially in East Africa (58·2 %).

**Table 3:** Relative frequency of people having experienced increasing prices in certain food groups, alcohol and cigarettes during the COVID-19 pandemic

	North Africa	West Africa	East Africa	Southern Africa	Western Europe	North America	Other	Total
Total (*n*)	136	251	146	83	172	105	136	1029
Cereals (%)	24·3	61·4	77·4	54·2	25·0	28·6	50·7	47·3
Dairy (%)	8·82	37·5	45·9	50·6	19·8	41·9	41·9	34·0
Fruits and veggies (%)	43·4	62·6	65·8	62·7	29·7	32·4	58·8	51·4
Meats (%)	27·2	53·4	50·0	62·7	24·4	61·9	47·1	45·4
Legumes (%)	28·7	52·2	67·1	47·0	16·9	18·1	40·4	39·8
Oils and fats (%)	14·0	39·8	52·1	54·2	14·0	18·1	34·6	32·1
Sweets (%)	9·56	36·3	50·7	51·8	14·0	12·4	39·0	30·2
Alcohol (%)	12·3	11·7	15·7	25·0	7·38	11·1	23·0	12·8
Cigarettes (%)	6·56	4·04	9·8	25·0	4·7	4·94	6·90	6·00

The restrictive security measures instituted during the early months of the pandemic have affected the dietary habits of the respondents. The most commonly reported issue was access to a lesser variety of foods (Table 4), particularly in North America (74·3 %) and East Africa (63·7 %). Food quality and quantity were also a common concern, especially in East Africa (51·4 and 61·6 %, respectively). In order to counteract the effect of the restrictive public health measures on their diet, people resorted to alternative approaches such as relying on other foods (49·2 %) or reducing portion sizes (30·3 %) (Table 4). These coping mechanisms were more frequent in African countries, especially East Africa where more respondents reported having to borrow food (32·9 %), reduce portion sizes (50·7 %), reduce the number of meals (45·9 %) and/or rely on food aid (18·5 %).

**Table 4:** Aspects of food insecurity experienced by the study participants and coping mechanisms adopted during the COVID-19 pandemic

	North Africa	West Africa	East Africa	Southern Africa	Western Europe	North America	Other	Total
Total (*n*)	136	251	146	83	172	105	136	1029
Aspects of food insecurity								
Difficulty accessing drinking water (%)	1·47	11·6	19·9	8·43	1·16	4·76	14·0	9·04
Lesser variety of food (%)	19·1	41·0	63·7	61·5	56·4	74·3	52·2	50·4
Food of less quality (%)	10·3	29·1	51·4	26·5	20·9	38·1	37·5	30·2
Lesser quantity of food (%)	8·82	35·9	61·6	51·8	40·7	49·5	33·8	39·2
Subject to food rationing (%)	6·62	14·3	19·2	27·7	7·56	7·62	16·9	13·6
Food less accessible (%)	16·2	23·9	39·0	15·7	12·8	25·7	20·6	22·3
Not having any food all day (%)	5·47	9·56	24·7	4·82	2·33	9·52	11·0	9·79
Eating less frequently (%)	19·5	23·1	45·9	31·3	11·1	29·5	26·5	25·7
Coping mechanisms								
Relying on less preferred food (%)	28·9	40·2	67·1	67·5	43·6	65·7	48·5	49·2
Borrowing food (%)	12·5	14·3	32·9	16·9	13·4	12·4	17·7	17·0
Food credit (%)	10·9	13·9	36·3	18·1	4·65	19·1	14·7	16·2
Reducing portion sizes (%)	19·5	28·3	50·7	34·9	19·8	31·4	31·6	30·3
Adults restricting themselves (%)	7·03	23·1	42·5	18·1	5·81	10·5	19·1	18·7
Reducing number of meals (%)	19·5	23·1	45·9	31·3	11·1	29·5	26·5	25·7
Food aid (%)	3·91	8·76	18·5	6·02	4·65	9·52	5·88	8·33

Vulnerable groups reported challenges in acquiring adequate foods (Table 5), especially people living with chronic diseases (20·2 %), the elderly (17·3 %) and children (14·5 %).

**Table 5:** Vulnerable groups whose diets have been affected by COVID-19 restrictions

	North Africa	West Africa	East Africa	Southern Africa	Western Europe	North America	Other	Total
Total (*n*)	136	251	146	83	172	105	136	1029
Infants	4·41	8·37	18·5	7·23	0·58	5·71	11·8	8·07
Children	0·15	15·9	35·6	15·7	2·91	6·67	18·4	14·5
Pregnant women	5·88	13·2	32·2	13·3	1·16	3·81	16·2	12·3
Breastfeeding women	2·94	12·4	30·1	9·64	0·58	3·81	13·2	10·7
Elderly	9·56	15·9	37·0	19·3	6·98	10·5	23·5	17·3
People with NCDs	10·3	19·9	41·1	24·1	11·1	15·2	21·3	20·2

## Discussion

In the current study, we documented the changes in food accessibility and availability globally as a result of the COVID-19 pandemic lockdowns which restricted the movement of people and goods. Specifically, we have shown that the lockdowns were accompanied by a reduced food diversity, a change in places where food was acquired and an increase in the stockpiling of cereals and other non-perishable foods (e.g. canned goods). In addition, our findings showed geographical disparities in the fluctuation of food prices and the nature of coping mechanisms during the COVID-19 pandemic.

Our study is one of a handful of studies documenting changes in food accessibility and availability during the early months of the COVID-19 pandemic and confirms the global impact of lockdowns on food availability and accessibility. Participants reported experiencing some forms of food insecurity, with over 50 % having access to lesser varieties of food, up to 74 % in North America. A study on the early impact of COVID-19 in the USA suggests that there was nearly a one-third increase (32·3 %) in household food insecurity due to COVID-19^(19)^. Food insecure participants experienced more challenges accessing food and utilising coping strategies^(19)^.

### Stockpiling of cereals and canned foods

The COVID-19 lockdowns triggered the shortages of staple foods due to bulk buying and stockpiling. Our study showed that, in all regions, respondents reported an increase in stockpiling cereals, legumes and canned products. This may lead to a greater consumption of processed and canned foods due to the unavailability or reduced affordability of healthier foods. A study in Ethiopia using macro-economic data showed that urban trade for high value, nutritionally rich foods such as fruits and vegetables was reduced. Additionally, post-harvest loss, market price increases and urban demand for nutritious commodities declined during the early days of the pandemic^(20)^. Consequently, dietary diversity declined. In Addis Ababa, for example, households were consuming less fruit (81 % declined to 60 %), meat (65 % declined to 54 %) and dairy (56 % declined to 45 %)^(21)^.

### Food prices

Participants reported changes in the price of food items, particularly those of fruits, vegetables, cereals and meat in African countries. Whilst it is uncertain how food prices will evolve should the pandemic persist to the end of 2021, it was predicted before COVID-19 that food imports may decline by 13 % at best and up to 25 % in the worst-case scenario^(22)^. This prediction was explained by higher transaction costs and reduced domestic demand. A decline in imports can lead to increased prices and a shortage of basic consumer goods, which may result in an increased inflation in some countries^(22)^.

### Dietary and lifestyle changes

Findings of this multi-country survey support previous findings of reported COVID-19-related dietary changes^(23)^. For example, Scarmozzino and Visioli^(23)^ reported that about 50 % of adult respondents modified their diet during the lockdown, with 46·1 % eating more and 19·5 % having gained weight. A global survey including 1047 respondents from Asia (36 %), Africa (40 %), Europe (21 %) and other regions (3 %) reported unhealthier food consumption patterns including changing types of food, compulsive eating, snacking between meals and increased number of main meals during lockdown^(24)^. Lockdowns may have resulted in an increased use of alcohol and other substances due to solitude. However, rules regarding the access and purchase of alcohol differed by country. For example, in South Africa, the lockdown included a ban on alcohol and cigarette sales. Interestingly, other studies reported a decrease in alcohol consumption^(23,25)^.

### Health implications

In the current study, some participants reported having trouble accessing drinkable water, eating less frequently and subject to reduced food diversity and quality. These changes can affect nutritional status, health and well-being^(26)^. Indeed, the nutritional status of individuals is associated with their capacity to resist infectious diseases; it is well known that the immune system is affected by malnutrition^(26)^. Nutrient intake has a definite relationship with antibody synthesis and the development of the immune system^(27)^. Consequently, a compromised nutritional status could lower the body’s ability to resist COVID-19 infection. Additionally, unhealthy dietary choices accompanied by a sedentary lifestyle could increase the incidence of non-communicable diseases. COVID-19 lockdowns have been associated with a more sedentary lifestyle^(28)^ which could increase the risk of obesity and related non-communicable diseases. However, a study conducted in Italy with 3533 participants reported that although 48·6 % of respondents perceived themselves as having gained weight, 38·3 % reported an increase in physical activity, especially body weight training, while 15 % of respondents reported purchasing organic fruits and vegetables and 3 % reported quitting smoking^(29)^. The overall health impact of COVID-19 lockdowns may only be apparent in the long-term.

### Policy implications

During crisis, the first action should be to create emergency livelihoods and food assistance intervention to guarantee food access to the most vulnerable. Emergency livelihoods is an adaptation of livelihood intervention to respond to the immediate need of the most vulnerable populations in times of crisis. Examples of interventions include emergency short-term employment/cash-for-work tailored to COVID-19 prevention measures such as building and rehabilitating sanitation of public areas, disinfection and cleaning of public buildings, provision of food assistance and financial grants for the most vulnerable households. COVID-19 has created new vulnerable groups such as casual workers, small business owners and private sector employees who lost their jobs and income. The list of beneficiaries of food and social assistance programmes should be adjusted to include these new vulnerable groups. The changes in food prices affect these new vulnerable groups; therefore, policies to improve access to nutritious foods are important. Additionally, as the COVID-19 pandemic persists, the provision of food assistance may not be sustainable. It is, therefore, important to promote long-term livelihood and resilience building solutions that will help the vulnerable to start economic activities and to sustain livelihoods and be self-sufficient.

Sustained progress towards achieving the sustainable development goals may help combat current and future crises^(30)^. The ongoing global agenda for food systems transformation, which aims to accelerate the achievement of sustainable development goals, should consider policy measures that facilitate immediate access to food for the most vulnerable and provide social protection programmes together with efficient food distribution in time of crisis. Governments should promote and develop capacities for innovative food distribution approaches such as the establishment of food hubs, mobile sales and e-commerce platforms or informal networks.

The rise in the price of the most nutritious food groups, such as fruits and vegetables, alongside reduced physical activity, due to home confinement and unemployment, may increase the prevalence of overweight and obesity and related non-communicable diseases. Access to accurate food price information should be prioritised as a policy response, in addition to instituting price control measures that aim to reduce the burden on the most affected. Further, governments should enhance food price monitoring and strengthen enforcement of violation of food price regulations. Food production, distribution and marketing should be monitored using rapid and repeated assessment tools in order to provide real-time information to evaluate the impacts on food security and inform post-COVID-19 recovery and management of future crises. The information can be disseminated through different channels including radio and social media. Such information provides opportunities for awareness campaigns on good food hygiene practices and nutritive value of different foods together with COVID-19 preventive measures.

Overall, while governments should be taking actions to mitigate the negative effects of restrictive measures on accessibility to nutritious food, maintaining social distancing measures will help prevent the food systems processes from becoming a source of propagation of the disease itself.

Our study has several strengths, including the use of empirical data and the ability to analyse data from diverse geographical regions. Nevertheless, the main limitation of our study is that our sample included mainly respondents with Internet access, high degrees and jobs, criteria that would somewhat constitute a middle class. Therefore, our results cannot be widely extrapolated beyond the scope of the current study. Interestingly, for some countries and regions, the fact that we observed high levels of food insecurity as a result of COVID-19 lockdowns among those in the population who are employed might be considered an indicator that the situation is probably worse in the lower economic strata of these populations. Also, it is likely that the food classifications used in the current study might not have the same definitions across countries. It is possible that the selection by the respondents of food items such as sweets or dairy may relate to diverse food items across countries. Likewise, other terms such as rural and urban may have different definitions across countries. Additionally, the country and regional differences in lockdown measures and food systems may mean populations may not experience these measures in the same manner and the coping mechanisms may also vary.

In conclusion, food insecurity has been recorded in all surveyed countries; participants reported struggling with insufficient and inadequate food. Alongside reduced physical activity due to home confinement and unemployment, there is a great risk that the repercussions might include an increased prevalence of non-communicable diseases and malnutrition. The data presented in the current study highlight the fragility of global food systems during the COVID-19 pandemic. There is an urgent need to rethink how we produce, process, market and consume our food to create more sustainable and resilient food systems that protect livelihoods in the face of vulnerabilities and crises, which may impact people’s ability to have access to healthier diets.

Subsequent surveys should also be planned in all countries in order to assess the impact of all the circumstances related to COVID-19 on populations’ health and nutritional status.
